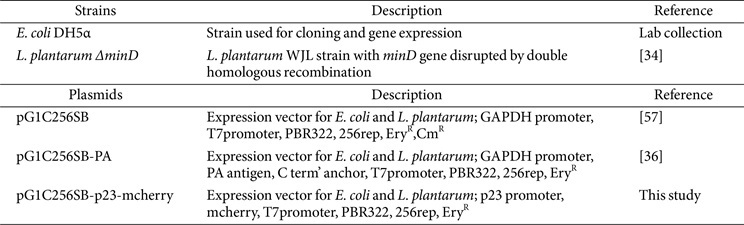# A Safe and Versatile Minicell Platform Derived from *Lactiplantibacillus plantarum* for Biotechnological Applications

**DOI:** 10.4014/jmb.2503.07031

**Published:** 2025-09-05

**Authors:** Junhyeon Park, Seungjune Chang, Heymin Kang, SangKu Yi, In-Hwan Jang, Kyung-Ah Lee, Donghyun Kim, Juhyun Kim

**Affiliations:** 1 School of Life Sciences, BK21 FOUR KNU Creative BioResearch Group, Kyungpook National University, Daegu 41566, Republic of Korea; 2 Department of Biomedical Sciences, Seoul National University College of Medicine, Seoul 03080, Republic of Korea; 3 Department of Microbiology and Immunology, Seoul National University College of Medicine, Seoul 03080, Republic of Korea; 4 Saeloun Bio Inc., Seoul 08826, Republic of Korea; 5 Institute of Endemic Diseases, Seoul National University Medical Research Center, Seoul 03080, Republic of Korea

**Keywords:** Minicell, *Lactiplantibacillus plantarum*, engineered bacteria, adjuvant, chemostat

## Abstract

Bacterial minicells are small and chromosome-free cells that result from aberrant cell division and represent a safe alternative to live microbial applications. However, most research on minicells has focused on *Escherichia coli*, with few studies exploring their development in non-model, biocompatible hosts. In this study, we engineered a *minD*-deficient *Lactiplantibacillus plantarum* (formerly *Lactobacillus arabinosus* and *Lactobacillus plantarum*) strain capable of producing minicells and systematically evaluated its potential as a chassis for biotechnological applications. Unlike *E. coli*-based systems, *L. plantarum* minicells exhibited stable accumulation of heterologous proteins and efficient surface antigen display without evidence of selective export or stress-induced release of toxic compounds. This behavior enabled uniform protein loading and consistent antigen presentation. Additionally, the minicells retained the immunostimulatory properties of their parent cells, underscoring their potential use as adjuvants *per se*. To improve production efficiency, we employed a continuous cultivation system with controlled growth conditions, which enabled steady-state operation and significantly enhanced minicell yield at optimal dilution rates. Collectively, these findings establish *L. plantarum*-derived minicells as a safe, robust, and genetically tunable platform suitable for therapeutic delivery, vaccine development, and immunoengineering.

## Introduction

Bacteria have been engineered to function as programmable chassis, enabling targeted and tunable delivery of therapeutic agents [1-3]. Their innate abilities to colonize specific tissues, penetrate physiological barriers, and respond to environmental cues make them attractive candidates for therapeutic applications, particularly in cancer therapy and infectious diseases [4-6]. By harnessing synthetic biology tools, these microbial platforms can be fine-tuned to control the expression, release, and localization of therapeutic molecules, thereby minimizing off-target effects and enhancing treatment efficacy [7-9]. For example, an *Escherichia coli* strain was engineered with an inducible secretion system to deliver therapeutic agents to tumors and recognize the epidermal growth factor receptor [10]. When applied, the programmed bacteria suppressed tumor growth *in vivo* and significantly prolonged host survival [10]. Additionally, these approaches allow microbes to serve as potent delivery vehicles for antigens, capable of stimulating robust and localized immune responses for vaccine development [11]. Their genetic tractability enables precise control over antigen expression, secretion [12], and surface display [13], while additional design elements—such as adjuvant production [14], and controllable lysis systems [15] —can further enhance immunogenicity.

Despite their therapeutic promise, the engineered living bacteria are constrained in clinical applications. Safety remains one of the foremost challenges in the clinical application of bacteria-based drug delivery. Even when using attenuated or non-pathogenic strains, the risk of uncontrolled bacterial proliferation or systemic infection persists, especially in immunocompromised patients or individuals with disrupted mucosal barriers [16, 17]. In addition, resource allocation in living bacteria may vary depending on the availability of certain nutrients to optimize cellular fitness, and genetic circuit programming often results in unintended outcomes due to unpredictable factors [18, 19]. These limitations highlight the need for safer and more robust alternatives that can maintain consistent payload levels regardless of culture conditions.

These limitations underscore the need for alternative platforms that are inherently safer and capable of maintaining consistent payload delivery regardless of environmental conditions. One promising solution lies in the use of bacterial minicells, which are small, anucleate vesicles naturally produced by certain bacterial strains as a result of aberrant cell division [20, 21]. Minicells lack chromosomal DNA, rendering them incapable of replication and thereby eliminating the risk of uncontrolled proliferation or horizontal gene transfer [20, 21]. Despite their genomic deficiency, minicells retain the necessary cellular machinery for gene expression, protein synthesis, and cargo packaging [22]. This makes them ideal candidates for programmable, non-replicating delivery vehicles that combine the functional advantages of living cells with a significantly improved safety profile. Due to their properties, minicells have been utilized in cancer therapy by encapsulating a wide range of chemotherapeutic and molecular agents, such as si/shRNA, antigens, and therapeutic toxins [20, 23-25]. Engineered minicells have shown potential in inhibiting the growth of drug-resistant tumors by effectively delivering the chemotherapeutics into hypoxic and necrotic regions in solid tumors [24, 25].

Such minicells can be intentionally produced by disrupting the cellular division system including the MinCD complex and MinE [26, 27]. Disruption of this system often leads to polar division events, resulting in the formation of minicells that lack chromosomal DNA, or, conversely, filamentous cells with multiple chromosomal copies [28, 29]. While the generation and utility of minicells have been widely studied in model bacteria such as *E. coli*, the development of minicell systems in non-model bacteria—such as Gram-positive *Lactiplantibacillus* species—remains largely unexplored. Expanding minicell technology to these non-model organisms is crucial as bacteria like *Lactiplantibacillus* are generally recognized as safe and have been used in food, probiotic, and therapeutic contexts, making them ideal candidates for both clinical and industrial applications. In addition, compared with existing delivery systems such as OMVs, liposomes, and SimCells, *L. plantarum*-derived minicells offer distinct advantages. Their rigid peptidoglycan cell wall and spherical morphology confer superior physical and chemical stability compared to lipid-based carriers [30, 31] and chromosome-free SimCells [32]. Moreover, with the available genetic information and engineering tools for *L. plantarum* [33], these minicells can be readily programmed to carry and/or sense diverse biomolecules. Owing to these properties, minicells derived from such chassis offer new opportunities for delivering therapeutic agents or metabolic products in environments where host safety and compatibility are critical. Accordingly, manipulating the division system of diverse bacteria to generate minicells allows us to design specialized minicell-based platforms tailored to specific biotechnological needs, including drug delivery and vaccine development.

In a previous study, we reported the generation of minicells from *L. plantarum* by deleting the *minD* gene [34]. In this current study, we further investigated the properties of these minicells, focusing on their capacity to accumulate heterologous proteins for potential biotechnological applications. Remarkably, the recombinant protein was evenly distributed throughout the cells, resulting in a level of protein accumulation in minicells comparable to their parent cells. Furthermore, when a surface display system was used to express a specific antigen in the *minD*-deficient strain, the antigen was successfully presented on the surface of the resulting minicells. Notably, these minicells also retained adjuvant activity comparable to that of the parental cells, highlighting their functional relevance in immunological applications. To enhance minicell production, we employed a chemostat-based cultivation strategy, which yielded a greater number of minicells than conventional batch cultures. Collectively, these complementary approaches enabled us to assess the functional potential of *L. plantarum*-derived minicells, establishing them as a robust and biocompatible platform for future therapeutic delivery and vaccine applications.

## Materials and Methods

### Culture Conditions

Unless otherwise indicated, *Escherichia coli* and *L. plantarum* strains were routinely cultured at 37°C in Luria-Bertani (LB) and Man–Rogosa–Sharpe (MRS) media, respectively. *E. coli* cultures were grown with shaking at 180 rpm, whereas *L. plantarum* cultures were incubated under static conditions. Erythromycin was added to cultures of bacterial cells as needed to ensure plasmid retention and maintenance of the manipulated genotype (200 μg ml^-1^ for *E. coli* and 5 μg ml^-1^ for *L. plantarum*). For experiments involving minicell production and protein expression analysis, the overnight cultures of *ΔminD* strain were diluted 1:100 in 100 ml of fresh medium. The cultures were then incubated for an additional 16 h to promote minicell production or protein accumulation. Minicells were subsequently isolated following the protocol described in a previous study [29, 34].

### Cloning Procedures and the Construction of Surface Display Strain

The characteristics of the bacteria and plasmids used in this study are described in Table 1. Plasmid DNA was isolated from bacterial cells using the Exprep Plasmid SV mini Purification kit (GeneAll, Republic of Korea). To construct an mCherry expression plasmid under the control of the strong constitutive P23 promoter and a synthetic GGGAGG ribosome binding site, a linear DNA fragment encoding the entire P23-RBS-mCherry cassette was synthesized (Macrogen, Republic of Korea). This DNA fragment was used as a template for PCR amplification using the primer pairs mCh-F; 5'-GGCGAATTGGCACCACTAGTCGAAAAGCCCTGACAACCCT-3' and mCh-R; 5'-ATAACCGATTCCCACCGCGGTCATTTATATAATTCGTCCA-3'. The amplicon was then joined by isothermal assembly [35] with pG1C256SB plasmid backbone, which was amplified using the primer pair (pG1C-F/R: 5'-AGGGTTGTCAGGGCTTTTCGACTAGTGGTGCCAATTCGCC-3' and 5'-TGGACG AATTATATAAATGACCGCGGTGGGAATCGGTTAT-3'). The resulting construct, designated as pG1C256SB p23::mCherry, was introduced into *E. coli* DH5α through electroporation, and positive clones were selected by assessing mCherry fluorescence in transformed colonies. The construct was subsequently introduced into *L. plantarum* *ΔminD* using an approach described previously [34]. Briefly, an overnight culture of *ΔminD* strain was prepared in 20 ml of MRS medium. A 2-ml sample was transferred into 48 ml of fresh MRS supplemented with 1% glycine and incubated until the culture reached an OD_600_ of ~0.6. Cell growth was halted by incubating on ice for 30 min. Cells were harvested by centrifugating at 6,000 rpm for 5 min and maintained at 4°C. The cell pellet was washed with 50 ml of cold 10 mM MgCl_2_ and centrifuged again. The supernatant was discarded, and the pellet was gently resuspended in 25 ml of cold SM buffer (900 mM sucrose, 3.5 mM MgCl_2_). The resulting pellet was used as competent cells, and electroporation was carried out to transfer the plasmid DNAs. Through this approach, we constructed *L. plantarum* *ΔminD* strain containing the PA expression vector [36].

### Measurement of Fluorescent Protein Expression

To evaluate the expression of the reporter protein, *L. plantarum* *ΔminD* strain harboring an mCherry-expressing plasmid were grown overnight in MRS medium and subsequently subcultured into 100 ml of fresh MRS until reaching the late exponential phase. The sample was then used to separate parent cells and minicell-enriched fractions, each sample was washed with PBS and adjusted to the same optical density at 600 nm. Fluorescence intensity was then measured using a microplate reader (Synergy H1, BioTek, USA) with excitation and emission wavelengths set at 595 nm and 620 nm, respectively. The samples were also observed using a fluorescent microscope.

### Analysis of the PA Surface Display

Surface-displayed protective antigen (PA) fused with an HA tag was analyzed through immunofluorescence staining. A 500 μl aliquot of an overnight bacterial culture was harvested by centrifugating at 9,000 rpm, and the cells were fixed in 4% paraformaldehyde for 30 min at room temperature (RT) with gentle rotation. After fixation, the cells were washed with PBS and blocked with 5% BSA in PBS for 1 h at RT. The samples were then incubated overnight at 4°C in the dark with a polyclonal anti-HA antibody (Thermo Fisher Scientific, USA), diluted 1:500 in 3% BSA/PBS. Subsequently, the samples were washed five times with 0.1% Triton X-100 in PBS and then incubated with Alexa Fluor-conjugated goat anti-rabbit IgG (Thermo Fisher Scientific), diluted 1:500 in 3% BSA/PBS, for 1 h at RT. Following five additional washes, the stained cells were resuspended in 200 μl of PBS for fluorescence analysis using either a NovoCyte flow cytometer (Agilent Technologies, USA) or a fluorescent microscope. For the flow cytometry analysis, a total of 50,000 events were collected per sample, and the mean fluorescence intensity of each population was quantified using the instrument’s native software. Bacterial imaging was conducted as previously described [29], with minor modifications. Briefly, samples were applied to coverslips pre-coated with poly-L-lysine (Sigma, USA) and allowed to air-dry at room temperature. The coverslips were then mounted onto slides with Prolong antifade reagent (Thermo Fisher Scientific) and sealed with clear nail polish. Fluorescence microscopy was performed using an Olympus BX53F2 microscope equipped with a 100× phase contrast objective and an FX900C camera. Alexa Flour 488, and mCherry channels were imaged using U-FFGFP, and U-FFTexas Red filter sets, respectively. Image processing was carried out using the ImageJ.

### Mice Immunization and Sample Collection

Eight-week-old C57BL/6 mice (OrientBio, Republic of Korea) were acclimatized to the animal facility for one week before use. The mice were immunized subcutaneously with 5 μg of human serum albumin (HSA; Sigma-Aldrich) diluted in 100 μl of sterile PBS (Gibco, USA) and mixed with either 100 μl of *L. plantarum*–derived minicells (~2 × 10^9^ cells per 100 μl) or an equal volume of sterile PBS, giving a total injection volume of 200 μl. Each mouse received two 100 μl injections using a sterilized 1 ml, 26-gauge syringe (Koreavaccine, Republic of Korea): one between the forelimbs and one between the hindlimbs. Serum samples were collected 14 days post-immunization for ELISA. All animal procedures were approved by the Institutional Animal Care and Use Committee of Seoul National University (Approval no. SNU-240711-3-1).

### Enzyme-Linked Immunosorbent Assay (ELISA) Analysis

Enzyme-linked immunosorbent assays (ELISA) were performed to quantify HSA-specific antibody responses. Briefly, 96-well ELISA plates (MaxiSorp; Nunc, USA) were coated overnight at 4°C with 1 μg of human serum albumin (HSA) per well in PBS, then sealed to prevent evaporation. Plates were washed three times with PBS containing 0.05% Tween-20 (PBST) and blocked for 1 h at room temperature with 1% (w/v) bovine serum albumin (BSA; Sigma-Aldrich) in PBS. Mouse sera were serially two-fold diluted in 1% BSA/PBS, and 100 μl of each dilution was added to the wells and incubated for 1 h at room temperature with gentle rocking. After three washes with PBST, mixture of alkaline-phosphatase (AP)-conjugated goat anti-mouse IgG_1_, IgG_2b_, IgG_2c_, and IgG_3_ (SouthernBiotech, USA) was added and incubated for 1 h at room temperature with gentle rocking. Plates were then washed five times, and bound antibodies were visualized by adding 100 μl of p-nitrophenyl phosphate (pNPP; 1 mg ml^-1^ in pNPP buffer, SouthernBiotech) for 30 min at room temperature. Absorbance was measured at 405 nm using a GloMax microplate reader (Promega, USA)

### Statistics

Statistical analyses were performed using GraphPad Prism (v10.2.0). Comparisons between two groups or multiple groups were made with a Student’s two-tailed *t* test or one-way ANOVA test, respectively. For comparison among multiple dilution factors were performed by two-way ANOVA with Tukey’s multiple comparisons test. P values lower than 0.05 were considered significant.

## Results and Discussion

### Minicell-Producing *L. plantarum* Maintains Robust Growth without Stress-Responsive Export

**Fig. 1:**
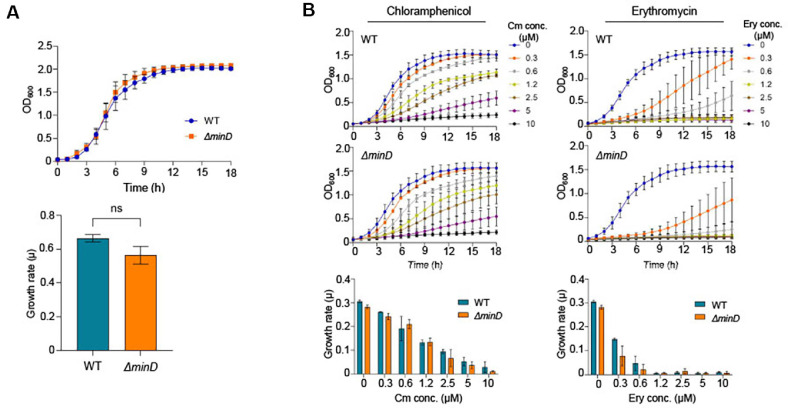
Comparative growth analysis of *L. plantarum* WT and *ΔminD* strains. (**A**) Growth curves of WT and *minD*-deficient *L. plantarum* cultured in MRS medium. Overnight cultures were diluted 1:100 into fresh MRS and inoculated into a 24-well plate. The cultures were incubated statically at 37°C, and their optical densities (OD_600_) were measured at a wavelength of 600 nm hourly for 18 h. Growth rates were calculated accordingly. Error bars represent mean ± SD (*n* = 3). (**B**) Growth responses of WT and *ΔminD* strains were evaluated under increasing concentrations of chloramphenicol (Cm) and erythromycin (Ery). Overnight cultures were diluted 1:100 into MRS medium supplemented with 0, 0.3, 0.6, 1.2, 2.5, 5, or 10 μM of each antibiotic and inoculated into a 24-well microplate. The cultures were incubated statically at 37°C, and OD_600_ was measured over 18 h using a microplate reader. Growth curves were generated to assess dose-dependent antibiotic sensitivity. Error bars indicate the mean ± SD (*n* = 3). ns, not significant.

We investigated whether the *minD*-deficient *L. plantarum* strain (*ΔminD*) exhibits altered resource allocation strategies compared to the wild-type (WT). To assess this, both the WT and *ΔminD* strains were cultured in Man–Rogosa–Sharpe (MRS) medium using 24-well microplates, and their growth was monitored using a microplate reader. The resulting growth curves revealed nearly identical kinetics between the two strains, showing no significant differences in lag phase, exponential growth rate, or final biomass yield (Fig. 1A). When growth was monitored in minicell-producing *E. coli*, a modest increase in doubling time was observed compared to the wild-type, likely due to the additional energy and time required for minicell formation [28]. In contrast, the *minD*-deficient *L. plantarum* strain exhibited no detectable growth defect under standard, non-stress conditions, despite its abnormal cell division phenotype. This suggests that the mutant strain maintains efficient resource allocation, supporting normal biomass production without significantly diverting resources toward minicell formation. These findings support the use of minicell-producing *L. plantarum* as an efficient platform for minicell engineering with predictable gene expression, as resource allocation remains largely unaffected by minicell production.

Previous studies have shown that minicell-producing *E. coli* can alleviate cellular toxicity of original cell by packaging and exporting antibiotics and toxic compounds within minicells [28, 37]. In this study, we investigated this phenomenon using the *minD*-deficient *L. plantarum* strain by growing it in the presence of sublethal concentrations of translation-inhibiting antibiotics, such as erythromycin and chloramphenicol. Unlike the behavior observed in *E. coli*, the minicell-producing *L. plantarum* strain did not exhibit increased fitness under these stress conditions (Fig. 1B). This suggests that, in *L. plantarum*, minicell formation is not employed as a mechanism to enhance cellular fitness by exporting toxic compounds or immature proteins. Instead, stress-inducing agents and misfolded proteins may remain evenly distributed throughout the cell population. Consequently, unlike in *E. coli*, minicell production in *L. plantarum* appears to preserve valuable intracellular content rather than eliminate it—highlighting its unique potential as a platform for accumulating useful enzymes or chemicals.

### Engineering *L. plantarum* Minicells for Intracellular Protein Accumulation and Surface Display of a Protective Antigen

**Fig. 2:**
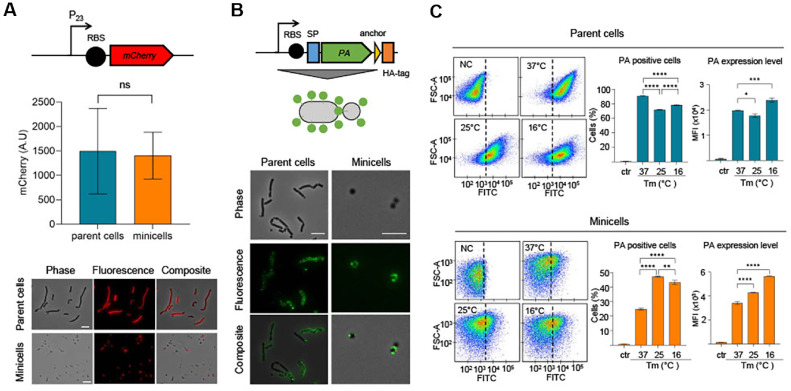
Functional analysis of *L. plantarum*-derived minicells. (**A**) Schematic representation of the plasmid construct pG1C256SB-p23-mCherry used for constitutive mCherry expression (upper panel). The reporter strain was cultured in MRS medium, and parent cells and minicells were separated. mCherry fluorescence was quantified from each fraction and normalized to equivalent cell mass (middle panel). Error bars represent the mean ± SD (*n* = 3). Representative fluorescence microscopy images are shown (scale bars, 5 μm; lower panel). (**B**) Schematic representation of the plasmid construct pG1C256SB-PA used for PA surface expression (upper panel). A protective antigen (PA) expression system was introduced into the *ΔminD* strain, and antigen localization and expression were assessed using immunofluorescence microscopy. The PA surface expression is schematically depicted in the middle panel; representative fluorescence images are shown in the lower panel (scale bars, 5 μm). (**C**) The strain was also cultured at different temperatures (16, 25, and 37°C), and the proportion of PA-positive cells was quantified through flow cytometry using the FITC channel. A strain lacking the PA expression system served as a negative control (NC). Left panel shows each representative plot under each condition. Right panel indicates the mean fluorescence intensity of each cells among PA-stained cells. Error bars represent the mean ± SD (*n* = 3). *P < 0.05, **P < 0.01, ***P < 0.001, ****P < 0.0001 by two-tailed Student’s t-test. ns, not significant.

Given the broad biotechnological potential of minicells, including their use in drug delivery systems, we next evaluated their capacity to accumulate a heterologous protein. To this end, we cloned an *mCherry* gene that was codon-optimized for Gram-positive bacteria under the control of the constitutive P_23_ promoter [38] on a p pG1C256SB plasmid, which was then introduced into the *ΔminD* strain (Fig. 2A). The reporter strain, capable of producing minicells, was cultured overnight in MRS medium, and the sample was used to separate parent cells and minicells. We then quantified the accumulation level of the fluorescent protein in each fraction using a microplate reader. We found that the OD-normalized mCherry fluorescence did not differ significantly between the parent cell and minicell fractions (Fig. 2A). This result indicates that minicells function as passive yet reliable carriers of intracellular content and could serve as minimal, genetically programmable platforms for protein-based applications. Although individual minicells accumulate lower amounts of heterologous proteins due to their smaller size compared to parental cells, the overall protein accumulation is comparable when equivalent biomass from each population is analyzed. This suggests that the fluorescent protein is evenly distributed throughout the cell population, without preferential localization or accumulation in specific subcellular compartments. Previous studies have shown that heterologous proteins often accumulate to higher levels in *E. coli*-derived minicells [29, 39], likely due to stochastic partitioning or selective export mechanisms [40-42]. While this enrichment can be advantageous for maximizing protein payloads, it limits control over the distribution of protein cargo among individual minicells, complicating predictable loading. In contrast, when *L. plantarum* was used as the host, the accumulation of heterologous protein was comparable between parent cells and minicells (Fig. 2A). This consistent distribution enables precise tuning of protein levels within minicells, supporting the use of *L. plantarum*-derived minicells as a reliable and scalable chassis for protein-based therapeutic delivery.

We also investigated whether minicells could support the surface display of functional proteins, with a particular focus on their potential use in vaccine applications. To this end, we employed a vector encoding a recombinant form of protective antigen (PA) fused to a surface display module and an HA tag, enabling its presentation on the cell surface as a potential vaccine platform against *Bacillus anthracis* [36, 43, 44]. We introduced the PA-expressing vector into the *ΔminD* strain and cultured the cells overnight in MRS medium at 37°C. The resulting culture was fractionated into parent cells and minicells, and each fraction was analyzed through immunofluorescence microscopy using an anti-HA antibody to assess antigen expression and localization. The results showed that fluorescent signals corresponding to PA were present in both parent cells and minicells (Fig. 2B). Notably, the signals were primarily localized on the cell surface, indicating that the antigen was successfully displayed as intended (Fig. 2B). While these findings confirm effective surface localization of PA in *L. plantarum*-derived minicells and demonstrate their inherent immunostimulatory effect in a co-immunization setting, it remains unclear whether this antigen display can trigger a PA-specific immune response. To address this, future studies will assess PA-specific humoral and cellular immunity *in vivo*, including antigen-specific IgG titers in a mouse model. In the present work, however, rather than evaluating antigen-specific immunity, we examined whether culture conditions influence the expression level of the antigen display system. To identify optimal conditions for surface display, we next compared the effect of culture temperature. Flow cytometry analysis revealed that only 25% of the purified minicells exhibited antigen-associated fluorescence at 37°C, whereas 91% of the parent cells showed positive signals (Fig. 2C). In contrast, culturing the PA-expressing *ΔminD* strain at lower temperatures (25°C and 16°C) resulted in an approximately two-fold increase in the proportion of antigen-positive minicells, while the positive population in parent cells decreased by about 20% (Fig. 2C). Moreover, minicells exhibited stronger PA expression at lower temperatures, indicating an inverse temperature-dependent trend. In contrast, PA expression in parent cells was highest at 16°C but decreased at 25°C, showing no consistent temperature-dependent pattern. (Fig. 2C). Although the underlying mechanism remains unclear, one plausible explanation is that lower temperatures promote the localization of the antigen–surface display complex near the cell poles, which are potential sites of minicell formation. This polar enrichment could enhance antigen display specifically in minicells, whereas parent cells without such localization enrichment may exhibit different temperature-dependent trends. Indeed, previous studies have reported that proteins fused with polar localization signals are enriched in minicells derived from such compartments [29, 37]. These observations suggest that *L. plantarum*-derived minicells have strong potential for vaccine development. Furthermore, optimization of culture conditions may be a key factor in enhancing antigen enrichment within minicells.

### Minicells Function as Adjuvants by Enhancing Immune Responses

**Fig. 3:**
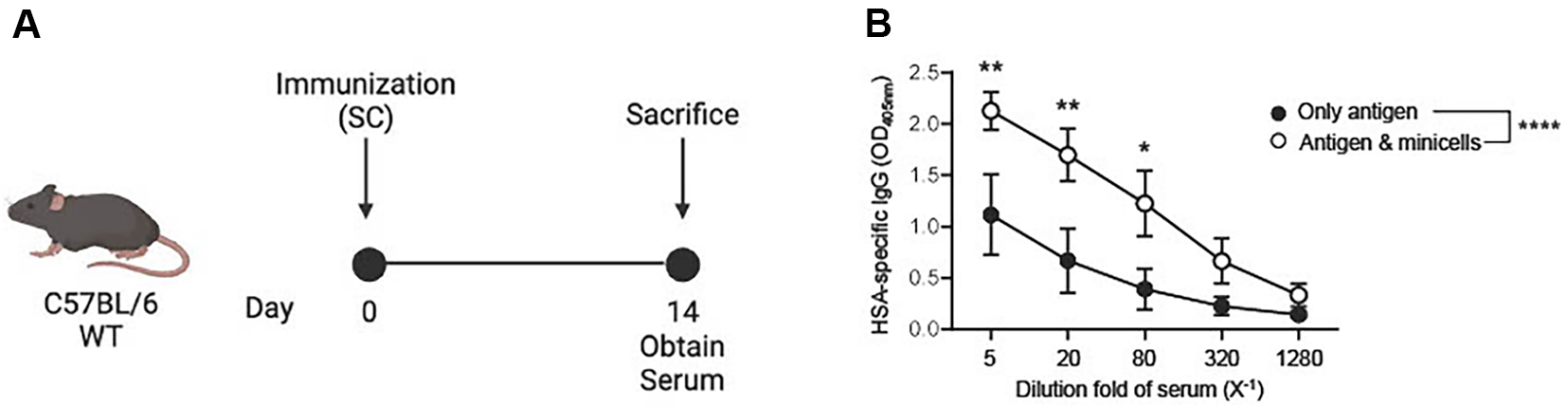
*L. plantarum* minicells exhibit adjuvant activities. (**A**) Schematic overview of the animal immunization protocol. A total volume of 200 μl was prepared for each immunization, which consisted of 5 μg human serum albumin (HSA) in 100 μl PBS mixed with either 100 μl PBS or 100 μl *L. plantarum* minicells. Each mouse received 100 μl of the mixture subcutaneously between the hindlimbs and another 100 μl between the forelimbs. (**B**) HSA-specific IgG levels in serum. HSAspecific IgG were measured in serum collected at 14 days after immunization. *P < 0.05, **P < 0.01, ****P < 0.0001 by two-way ANOVA with Tukey’s multiple comparison.

Previous studies have reported that certain strains of *Lactiplantibacillus* exhibit intrinsic adjuvant properties, enhancing both innate and adaptive immune responses when co-administered with antigens [45]. This effect is largely attributed to their Gram-positive cell wall structure, which contains immunostimulatory components such as lipoteichoic acids (LTA), peptidoglycans, and surface-layer (S-layer) proteins. These microbial-associated molecular patterns are recognized by host pattern recognition receptors, including Toll-like receptors (TLRs) and NOD-like receptors (NLRs), thereby triggering cytokine production, dendritic cell activation, and antigen presentation [45, 46]. In this study, we hypothesized that *L. plantarum*-derived minicells, which retain the parental cell-wall structure, could likewise function as adjuvants. To verify this hypothesis, we subcutaneously immunized C57BL/6 mice with human serum albumin (HSA; as an antigen) either alone or in combination with *L. plantarum* minicells. Fourteen days later, the animals were euthanized, and their sera were collected (Fig. 3A). Co-administration of minicells significantly increased HSA-specific IgG production compared with HSA alone (Fig. 3B), demonstrating that minicells could potentiate systemic antibody responses. Importantly, the adjuvanticity of *Lactiplantibacillus* species is generally associated with a favorable safety profile due to their long-standing use as probiotics and their classification as GRAS (Generally Recognized As Safe) organisms. Compared to Gram-negative bacteria such as *Salmonella* or *E. coli*, which carry lipopolysaccharide (LPS) in their outer membranes and may elicit excessive or uncontrolled inflammation, *Lactiplantibacillus*-based systems offer a safer alternative for vaccine applications. LPS is a potent endotoxin that can induce systemic toxicity, often necessitating extensive genetic modifications or detoxification strategies in Gram-negative vaccine platforms [47, 48]. LPS can constitute up to 75% of the outer membrane in Gram-negative bacteria such as *Salmonella* and *E. coli* [49]. In contrast, *Lactobacillus*-derived minicells, lacking LPS due to their Gram-positive cell wall architecture, can stimulate immune responses without the risk of LPS-mediated endotoxicity. Therefore, *Lactiplantibacillus*-derived adjuvants activate immune responses without such risks, making them attractive candidates for mucosal and parenteral immunization. Furthermore, since minicells are incapable of replication [20, 21], *L. plantarum*-derived minicells are expected to serve as a safer and effective adjuvant system that maintains the immunostimulatory features of their parental cells. Future studies will focus on expanding the potential applications of *L. plantarum*-derived minicells — for example, by enabling surface antigen expression and establishing a more robust evidence base for their application as safe and effective vaccine adjuvants.

### Optimization of Minicell Production from *L. plantarum* Using a Growth-Controlled Continuous Culture System

**Fig. 4:**
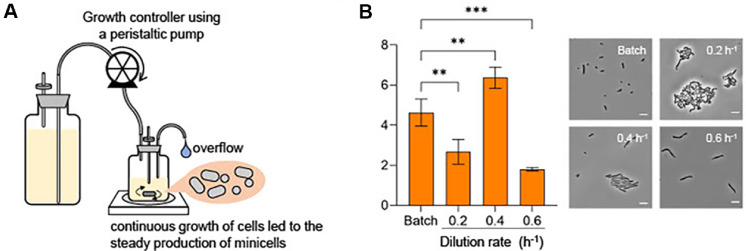
Optimization of minicell production through growth control in a continuous culture system. (**A**) Schematic illustration of the continuous culture setup. This system enables control of cellular growth rate by adjusting the dilution rate, allowing cells to maintain division under physiologically steady-state conditions. (**B**) Minicell production according to different dilution rates. Minicells were collected from equal volumes of culture broth grown under each condition. The number of obtained minicells were quantified using flow cytometry (left panel) and their morphologies were observed via bright field microscopy (right panel, each representative image). Error bars represent mean ± SD (*n* = 3). **P < 0.01, ***P < 0.001 by one-way ANOVA test.

In order to enhance their practical utility, it is essential to optimize production efficiency of minicells. To this end, we employed a minimized chemostat cultivation approach [49], which enables precise control of growth parameters and supports continuous cellular growth, thereby maintaining constant cellular division and sustained minicell productions (Fig. 4A). In a chemostat system, fresh medium is continuously supplied while spent culture is simultaneously removed. This setup allows for tight regulation of cellular growth by adjusting the dilution rate of the culture medium [50-52]. The *L. plantarum* *ΔminD* strain was cultured in a chemostat at defined growth rates (0.2, 0.4, and 0.6 h^-1^), and the resulting minicell production was quantified. After five generations of growth, the cultures reached a physiologically steady state (data not shown) [52, 53]. We prepared minicells from equal culture volumes across all conditions using sequential centrifugation and filtration steps as described previously [29, 34]. This procedure effectively removed most parental cells, enabling accurate quantification of the minicell fraction. The number of minicells was quantified using flow cytometry. Interestingly, minicell production peaked at the intermediate growth rate, showing greater yield than those obtained under batch culture condition (Fig. 4B). In contrast, lower levels of minicell production were observed at both 0.2 h^-1^ and 0.6 h^-1^ (Fig. 4B). At 0.2 h^-1^, cells may have experienced stationary phase-associated stress, as most parent cells appeared curved under phase-contrast microscopy (Fig. 4B). Although no changes in cellular morphology were observed at 0.6 h^-1^ (Fig. 4B), this condition appears to impose excessive dilution pressure, likely exceeding the strain’s maximal growth capacity in a chemostat setting. These results show that minicell production can be maximized by precisely tuning the growth flow rate through optimal chemostat parameters. Importantly, the Introduction of genetic circuits into minicell-producing strains may alter their physiological properties, including growth behavior [54-56]. Therefore, fine-tuning dilution rate remains critical for maintaining optimal minicell production condition. This chemostat-based strategy provides a robust and scalable platform for maximizing yields of engineered minicells, further supporting their application in therapeutic and biotechnological systems.

## Conclusion

In this study, we developed and characterized a *L. plantarum*-derived minicell platform with broad potential for safe and programmable biotechnological applications. Using a *minD*-deficient strain, we confirmed the generation of minicells, consistent with observed in *E. coli*. However, *E. coli*-based systems, *L. plantarum* minicells did not appear to selectively export toxic compounds, highlighting their stability and suitability for enriching valuable biomolecules rather than disposing of cellular waste. These minicells also demonstrated the capacity to uniformly accumulate heterologous proteins and present surface antigens, underscoring their utility for intracellular protein delivery as well as vaccine development. Importantly, we found that *L. plantarum* minicells retained immunostimulatory properties, like their parent cells, indicating their potential use as adjuvants *per se* in immunological applications. To further improve production yield, we employed a chemostat-based cultivation system with controlled growth parameters, enabling stable and enhanced minicell generation under defined conditions. These results underscore the importance of precise growth control in optimizing minicell production, particularly when synthetic circuits or therapeutic payloads are introduced. Taken together, our findings establish *L. plantarum* minicells as a safe, genetically programmable, and functionally versatile platform suitable for therapeutic delivery, vaccine development, immune modulation, and a wide range of biotechnological applications.

## Figures and Tables

**Table 1:** Bacterial strains, plasmids used for this study.